# Hyperechoic mesenteric fat on preoperative intestinal ultrasound predicts early postoperative recurrence in Crohn’s disease

**DOI:** 10.1186/s13244-026-02251-2

**Published:** 2026-04-16

**Authors:** Zhuohua Liang, Shuting Chen, Wenjie Cheng, Si Qin, Xiaofang Hong, Xueting Guan, Jie Zhou, Guangjian Liu

**Affiliations:** 1 Department of Medical Ultrasound, Biomedical Innovation Center, the Sixth Affiliated Hospital, Sun Yat-sen University, Guangzhou, China; 2 Department of Radiology, Guangdong Provincial People’s Hospital (Guangdong Academy of Medical Sciences), Southern Medical University, Guangzhou, China; 3 Department of Radiology, the Sixth Affiliated Hospital, Sun Yat-sen University, Guangzhou, China

**Keywords:** Crohn’s disease, Early postoperative recurrence, Intestinal ultrasound, Hyperechoic mesenteric fat

## Abstract

**Objective:**

To investigate the value of hyperechoic mesenteric fat (HMF) detected by preoperative intestinal ultrasound (IUS) in predicting early postoperative recurrence (EPR) in patients with Crohn’s disease (CD).

**Materials and methods:**

This retrospective study included 124 CD patients who underwent I-stage intestinal resection. Based on 1-year postoperative recurrence, patients were stratified into EPR (*n* = 59) and non-EPR (*n* = 65) groups. Clinical parameters (such as smoking history, C-reactive protein (CRP), erythrocyte sedimentation rate (ESR), immunosuppressive therapy) and IUS parameters (such as bowel wall thickness (BWT), HMF, abscess/fistula) were compared. Univariate and multivariate logistic regression identified EPR predictors.

**Results:**

EPR occurred in 59 patients (47.6%) during the 1-year follow-up. Significant differences (*p* < 0.05) were observed between EPR and non-EPR groups for clinical factors (smoking history, elevated preoperative CRP/ESR, postoperative immunosuppression) and IUS parameters (HMF, BWT, and abscess/fistula). HMF demonstrated superior discriminative capacity for EPR prediction (area under the curve (AUC) = 0.808, 95% confidence interval (CI): 0.728–0.873) vs BWT (AUC = 0.618, *p* < 0.05) and abscess/fistula (AUC = 0.599, *p* < 0.05). Univariate analysis identified CRP, ESR, colonic disease, immunosuppression, HMF, abscess/fistula, and BWT as candidate predictors (*p* < 0.05), with multivariate analysis confirming HMF as an independent predictor (adjusted odds ratio (OR) = 18.810, 95% CI: 6.459–54.775; *p* < 0.001).

**Conclusions:**

Preoperative IUS-detected HMF may serve as a valuable predictor for assessing EPR risk in patients with CD.

**Critical relevance statement:**

Preoperative IUS identification of HMF provides a practical, non-invasive biomarker that enables radiologists to critically improve risk stratification for EPR in CD.

**Key Points:**

- EPR poses a significant clinical challenge in the management of CD.
- HMF detected on preoperative IUS is a strong, independent predictor for EPR, with superior predictive performance compared to other established IUS parameters.
- Assessing HMF provides a valuable, non-invasive imaging biomarker for preoperative risk stratification.

**Graphical Abstract:**

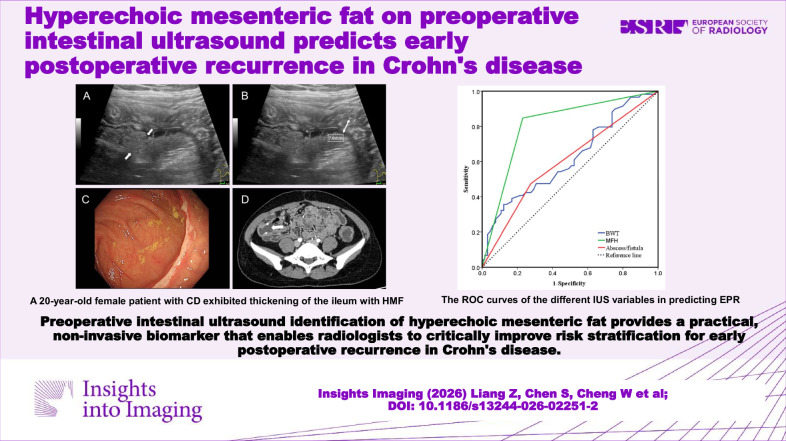

## Introduction

Crohn’s disease (CD) is an idiopathic, progressive, and chronic inflammatory condition that can affect any segment of the gastrointestinal tract, characterized by its unpredictable disease course, approximately 60%–80% of patients with CD will require surgical intervention during their lifetime due to associated complications [1]. After ileal or ileocolonic resection, 30%–90% of patients will experience early postoperative recurrence (EPR) within the first year [2, 3], EPR was defined as the recurrence occurring within 10 cm proximal or distal to the anastomosis site within one year following surgery [4]. Postoperative recurrence is an important reason why CD is regarded as an incurable benign disease. Monitoring and treating EPR is essential to prevent repeated surgery or severe complications in patients with CD [5].

Several clinical factors have been identified as potential predictors of EPR in CD, including age, smoking status, perforating phenotype, pharmacological treatment, and the length of the resected bowel [5]. The presence of inflammatory mesenteric fat (IMF), characterized by hypertrophy and chronic inflammation, has been recognized as a typical pathological feature of CD [6]. Emerging evidence suggests that the IMF significantly influences the course of CD through its immunomodulatory and endocrine functions [7]. Furthermore, while the IMF is strongly correlated with intestinal transmural inflammation, its role in causing EPR remains unclear [8, 9].

Though the IMF is a crucial factor in disease management and prognosis, its assessment is typically limited to direct pathological observation or surgical evaluation. In contrast, intestinal ultrasound (IUS) offers a non-invasive means to visualize and evaluate the mesentery. Hyperechoic mesenteric fat (HMF) is a key sonographic sign that is thought to reflect the underlying pathological changes of IMF. Accordingly, this study aims to investigate whether preoperative IUS-detected HMF can serve as a reliable predictor of EPR in patients with CD.

## Materials and methods

### Ethical considerations

This study was approved by the institutional ethics review board at the Sixth Affiliated Hospital, Sun Yat-sen University (no. 2024ZSLYEC-337). Due to the retrospective design of the study and the use of de-identified data, informed consent from individual participants was waived by the ethics committee. Confidentiality and privacy of the participants’ data were rigorously maintained throughout the study.

### Study population

This was a single-center, retrospective study that included data on consecutive patients with CD. The inclusion criteria were as follows: (a) patients diagnosed with CD based on the third consensus of the European Crohn’s and Colitis Organization (ECCO) guidelines [10], (b) availability of preoperative IUS within 3 months of surgery, and (c) availability of follow-up data, including endoscopy or cross-sectional imaging, one year after surgery. The exclusion criteria were as follows: (a) patients who underwent enterostomy instead of I-stage anastomosis, (b) insufficient evaluation of the mesentery surrounding the resected intestinal segment by IUS or inadequate imaging quality due to factors such as pregnancy or obesity (body mass index > 30 kg/m^2^), and (c) insufficient clinical data.

**Fig. 1:**
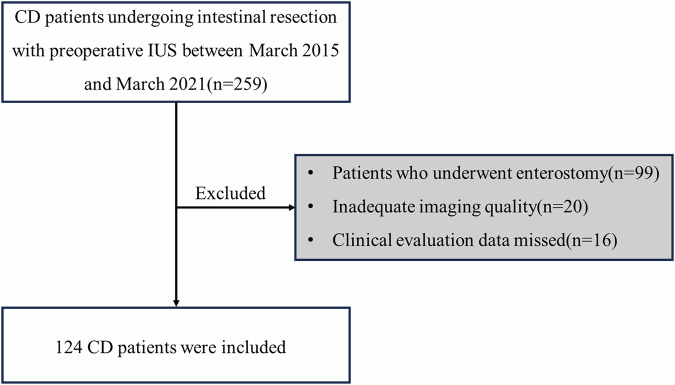
Flow diagram of the study population. CD, Crohn’s disease; IUS, intestinal ultrasound

A total of 259 patients were eligible for inclusion in the study, who underwent intestinal resections due to bowel damage (such as stenosis, fistulas, bleeding, and adhesions) between March 2015 and March 2021. However, 135 patients who met the exclusion criteria were subsequently excluded, including 99 patients who underwent enterostomy, 20 patients with inadequate imaging quality, and 16 patients who had missing clinical evaluation data. Therefore, 124 patients (80 males and 44 females; mean age, (31.60 ± 10.57 years; range, 14–66 years) were enrolled in the study. The study flowchart is shown in Fig. 1.

Patients were reviewed every 3–6 months following surgery, with the follow-up endpoint set at 12 months. Endoscopy and radiological assessments have been established as an effective modality for assessing postoperative recurrence [4, 11]. Both radiological and endoscopic recurrence served as diagnostic criteria in this study. Either modality sufficed for establishing recurrence; however, endoscopic confirmation took precedence when findings from both methods were available. EPR was defined as disease recurrence within 10 cm (proximal or distal) of the anastomotic site within one year after surgery, including recurrence at the anastomosis itself or within the adjacent 10 cm segment [4]. Endoscopic recurrence corresponded to a modified Rutgeerts score of ≥ i2b [4], while radiological recurrence was identified by imaging features of active CD at the anastomotic site (e.g., abnormal bowel wall enhancement, stenosis, intestinal fistula, and the comb sign) [4, 11]. Patients were defined as non-EPR if all imaging and endoscopic examinations were negative at the end of follow-up. The patients were divided into the EPR group (*n* = 59) and the non-EPR group (*n* = 65) according to whether recurrence occurred within 1 year after operation.

### Clinical information collection

The following baseline clinical characteristics were collected: sex, age at surgery, time interval from CD diagnosis to surgery (months), time interval from preoperative IUS to surgery (days), surgical anastomosis type, the length of the resected bowel, body mass index (BMI), smoking history, type of disease, behavior of disease, perianal disease, presence of previous intestinal resections for CD, peri-operative C-reactive protein (CRP), peri-operative erythrocyte sedimentation rate (ESR), preoperative platelet (PLT), peri-operative albumin (ALB), and the use of medications, including 5-aminosalicylic acid (5-ASA), steroids, immunosuppressants, and biologic agents. For medication use, preoperative use included the 3 months prior to surgery, while postoperative use encompassed the period from surgery to recurrence or up to 1 year after the operation in non-recurrent patients.

### IUS examination

All the IUS examinations were conducted by experienced radiologists with 5 to 8 years of experience in IUS. All the examinations were performed using the uniform equipment (LOGIQ E9; GE Healthcare). Initially, low-frequency curved array transducers (frequency range 3.0–5.0 MHz) were used for detecting inflammation, as well as abdominal effusion. Subsequently, a high-resolution linear array probe (frequency range 8.4–11.0 MHz) was employed to conduct a detailed examination of the bowel wall and mesenteric fat. IUS assessed the jejunum, ileum, right colon, transverse colon, and left colon. The following information was obtained from the affected segments and recorded in an electronic case report form: disease location, bowel wall thickness (BWT), color doppler signal (CDS) of bowel flow, bowel wall stratification (BWS), HMF, enlarged mesenteric lymph nodes, and complications like abscess/fistula [4, 11]. HMF is defined as homogeneous, hyperechoic changes around the bowel wall, and it is different in appearance from the striated, intermixed echoes of normal mesentery [6]. Mesenteric lymphadenopathy was defined as a short diameter ≥ 5 mm [11].

### Image analysis

Two radiologists (Z.L. and W.C.), who were blinded to the clinical data and the postoperative outcome, reviewed IUS images independently. Inter-observer disagreement was resolved and reassessed by G.L. (with 10 years of experience in intestinal imaging).

According to the anatomical markers on the surgical records, the location of the resected intestinal segment was determined. BWT was calculated as the average of four measurements: two along the long axis and two along the transverse axis. BWS was graded on a scale of 0 (consistent visualization of at least three distinct wall layers), 1 (focal disappeared), or 2 (extensive disappeared). The Limberg grading system is divided into five grades: Grade 0 (BWT ≤ 3 mm); Grade I (BWT > 3 mm without abnormal blood flow signals detected); Grade II (BWT > 3 mm with spotty or short striped blood flow signals); Grade III (BWT > 3 mm with long striped (> 1 cm) blood flow signals visible); and Grade IV (BWT > 3 mm with long striped blood flow signals connected to the mesentery.

### Statistics analysis

All statistical analyses were conducted using SPSS (Version 22.0, IBM Corporation). Categorical data are presented as *n* (%) and were compared using the χ² test or Fisher’s exact test, as appropriate. Normality of continuous data was assessed. Data conforming to a normal distribution are expressed as mean ± standard deviation (SD) and were compared between groups using the independent samples *t*-test. Non-normally distributed data are expressed as median (Q1, Q3) and were compared between groups using the Mann–Whitney *U*-test. IUS parameters and clinical characteristic variables were included in univariate analysis. IUS parameters demonstrating statistically significant differences in the univariate analysis were used to plot receiver operating characteristic (ROC) curves to evaluate diagnostic performance. IUS parameters and clinical characteristic variables that showed statistically significant differences in the univariate analysis were subsequently entered into a binary multivariable logistic regression analysis to identify independent predictors of EPR. For interobserver agreement assessment, Cohen’s kappa or weighted kappa was used for categorical variables and the intraclass correlation coefficient for continuous variables. A two-sided *p* value < 0.05 was considered statistically significant.

## Results

### Comparison of baseline clinical characteristics between the EPR and non-EPR groups

During the 1-year postoperative follow-up, EPR occurred in 47.6% (59/124) of CD patients. Diagnosis was confirmed by endoscopy in 98 patients and by follow-up imaging examinations in 26 patients. Among the analyzed clinical factors, statistically significant differences (all *p* < 0.05) were observed between the EPR and non-EPR groups regarding history of smoking, preoperative CRP and ESR levels, and postoperative immunosuppressive therapy. No statistically significant differences (all *p* > 0.05) were identified for the remaining parameters, as detailed in Table 1.

**Table 1:** Comparison of clinical characteristics between the EPR and Non-EPR groups in CD patients

Variables	Non-EPR group (*n* = 65)	EPR group (*n* = 59)	*p* value
Gender (%)			0.139
Male	38 (58)	42 (71)	
Female	27 (42)	17 (29)	
Age, years [*M* (*Q*_1_, *Q*_3_)]	32 (26, 36.5)	27 (22, 36)	0.086
Time from CD diagnosis to surgery, months [*M* (*Q*_1_, *Q*_3_)]	51 (19.5, 108)	60 (19, 96)	0.636
Time from preoperative IUS to surgery, days [*M* (*Q*_1_, *Q*_3_)]	13 (10, 20)	13 (8, 20)	0.731
BMI, kg/m^2^ [*M* (*Q*_1_, *Q*_3_)]	18.60 (17.62, 20.46)	18.38 (17.10, 20.00)	0.316
Type of surgical anastomosis (%)			0.194
Side-side	55 (85)	42 (71)	
End-side	4 (6)	7 (12)	
End-end	6 (9)	10 (17)	
Behavior of disease (%)			0.293
Non-stricturing and non-penetrating	16 (25)	8 (14)	
Stricturing	22 (34)	22 (37)	
Penetrating	42 (41)	29 (49)	
Perianal disease (%)			0.844
Yes	32 (49)	28 (48)	
No	33 (51)	31 (52)	
Smoking history (%)			0.029
Yes	6 (9)	0 (0)	
No	59 (91)	59 (100)	
Type of disease (%)			0.080
Ilea	24 (37)	18 (31)	
Colonic	3 (5)	10 (17)	
Ileocolonic	38 (58)	31 (52)	
Previous intestinal resection (%)			0.464
Yes	8 (12)	10 (17)	
No	57 (88)	49 (83)	
length of resected diseased bowel segment, cm, [*M* (*Q*_1_, *Q*_3_)]	30 (22, 50.5)	27 (14, 46)	0.115
Preoperative CRP, mg/L, [*M* (*Q*_1_, *Q*_3_)]	2,94 (0.86, 10.73)	18.60 (2.47, 68.42)	< 0.001
Preoperative ESR, mm/h, [*M* (*Q*_1_, *Q*_3_)]	16.0 (8.0, 31.0)	28.00 (14.00, 40.00)	0.011
Preoperative PLT, ×10^9^/L, [*M* (*Q*_1_, *Q*_3_)]	236.0 (201.5, 285.0)	228.00 (201.00, 271.60)	0.789
Preoperative albumin, g/L, [*M* (*Q*_1_, *Q*_3_)]	38.98 (35.67, 42.67)	37.84 (33.59, 41.29)	0.153
Preoperative medications (%)			
5-ASA			0.963
Yes	13 (20)	12 (20)	
No	52 (80)	47 (80)	
Steroid			0.604
Yes	11 (17)	8 (14)	
No	54 (83)	51 (86)	
Immunosuppressants			0.239
Yes	23 (35)	27 (46)	
No	42 (65)	32 (54)	
Biologics			0.096
Yes	8 (12)	14 (24)	
No	57 (88)	45 (76)	
Postoperative medications (%)			
5-ASA			0.069
Yes	8 (12)	2 (3)	
No	57 (88)	57 (97)	
Steroid			0.108
Yes	2 (3)	6 (10)	
No	63 (97)	53 (90)	
Immunosuppressants			0.020
Yes	38 (58)	46 (78)	
No	27 (42)	13 (22)	
Biologics			0.188
Yes	33 (51)	23 (39)	
No	32 (49)	36 (61)	

*Non-EPR* non-early postoperative recurrence, *EPR* early postoperative recurrence, *CRP* C-reactive protein, *ESR* erythrocyte sedimentation rate, *PLT* platelet, *5-ASA* 5-aminosalicylic acid

### Comparison of preoperative IUS parameters between EPR and non-EPR groups and ROC curve analysis results

Overall, inter-observer agreement for the sonographic features was good to excellent for most IUS parameters (e.g., kappa value = 0.886 for HMF), as detailed in Supplementary Table 1. Statistically significant differences (all *p* < 0.05) were observed between the EPR group and the non-EPR group in preoperative HMF, BWT, and abscess/fistula. No statistically significant differences were found for the other IUS parameters assessed (all *p* > 0.05; Table 2).

**Table 2:** Comparison of IUS parameters between the EPR and non-EPR groups in CD patients

Variables	Non-EPR group (*n* = 65)	EPR group (*n* = 59)	*p* value
BWT, mm, [*M* (*Q*_1_, *Q*_3_)]	6.6 (5.2, 7.5)	7.0 (5.8, 9.7)	0.024
BWS (%)			0.129
Preserved	27 (41)	15 (25)	
Focal disappeared	27 (41)	28 (48)	
Extensive disappeared	11 (18)	16 (27)	
Limberg classification (%)			0.698
Grade 0	0 (0)	1 (2)	
Grade I	2 (4)	2 (4)	
Grade II	28 (42)	20 (34)	
Grade III	29 (50)	31 (52)	
Grade IV	6 (4)	5 (8)	
HMF(%)			< 0.001
No	59 (77)	8 (14)	
Yes	15 (23)	51 (86)	
Abscess/fistula (%)			0.023
No	47 (72)	31 (52)	
Yes	18 (28)	28 (48)	
Mesenteric lymphadenopathy (%)			0.078
No	53 (81)	40 (68)	
Yes	12 (19)	19 (32)	
Abdominal effusion (%)			0.337
No	63 (97)	55 (93)	
Yes	2 (3)	4 (7)	

*BWT* bowel wall thickness, *BWS* bowel wall stratification, *HMF* hyperechoic mesenteric fat

**Fig. 2:**
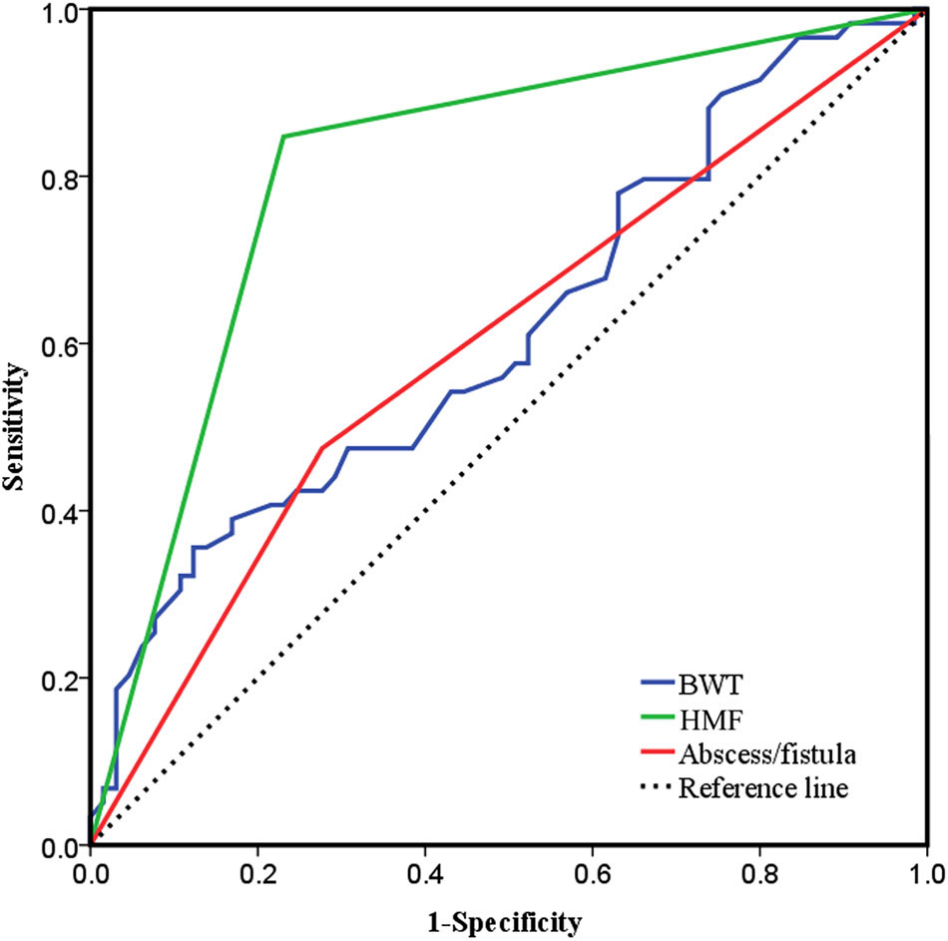
The ROC curves of the different IUS variables in predicting EPR

**Fig. 3:**
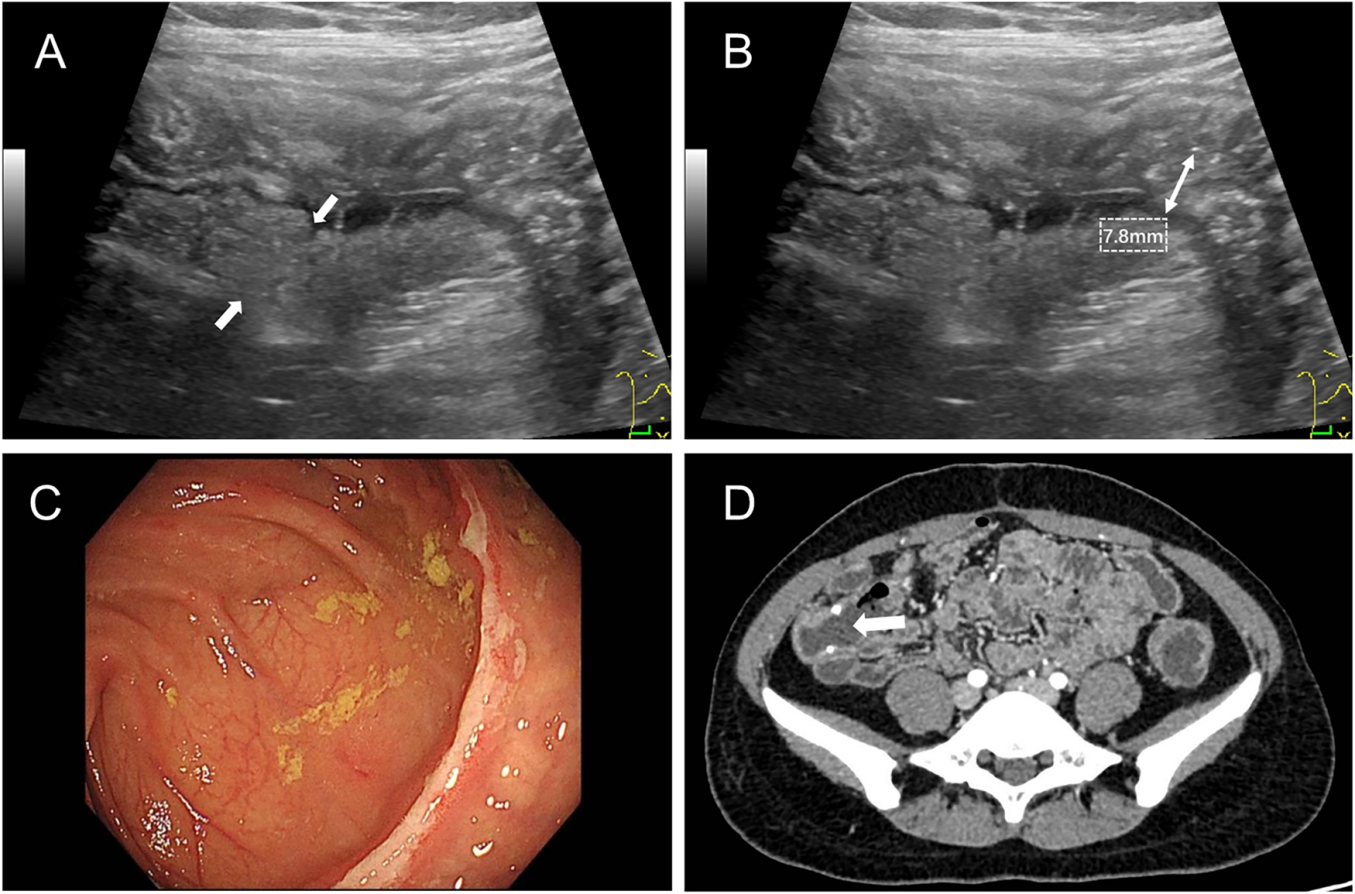
A 20-year-old female patient with CD exhibited thickening of the ileum with HMF (**A**, solid arrow). The thickness of the diseased bowel was measured at 7.8 mm (**B**). Eight months after an ileocecectomy, inflamed mucosa with ulcers was detected during endoscopy (**C**), and a CT scan showed that the bowel wall at the anastomosis (hollow arrow) was thickened (**D**). CD, Crohn’s disease; HMF, hyperechoic mesenteric fat

**Fig. 4:**
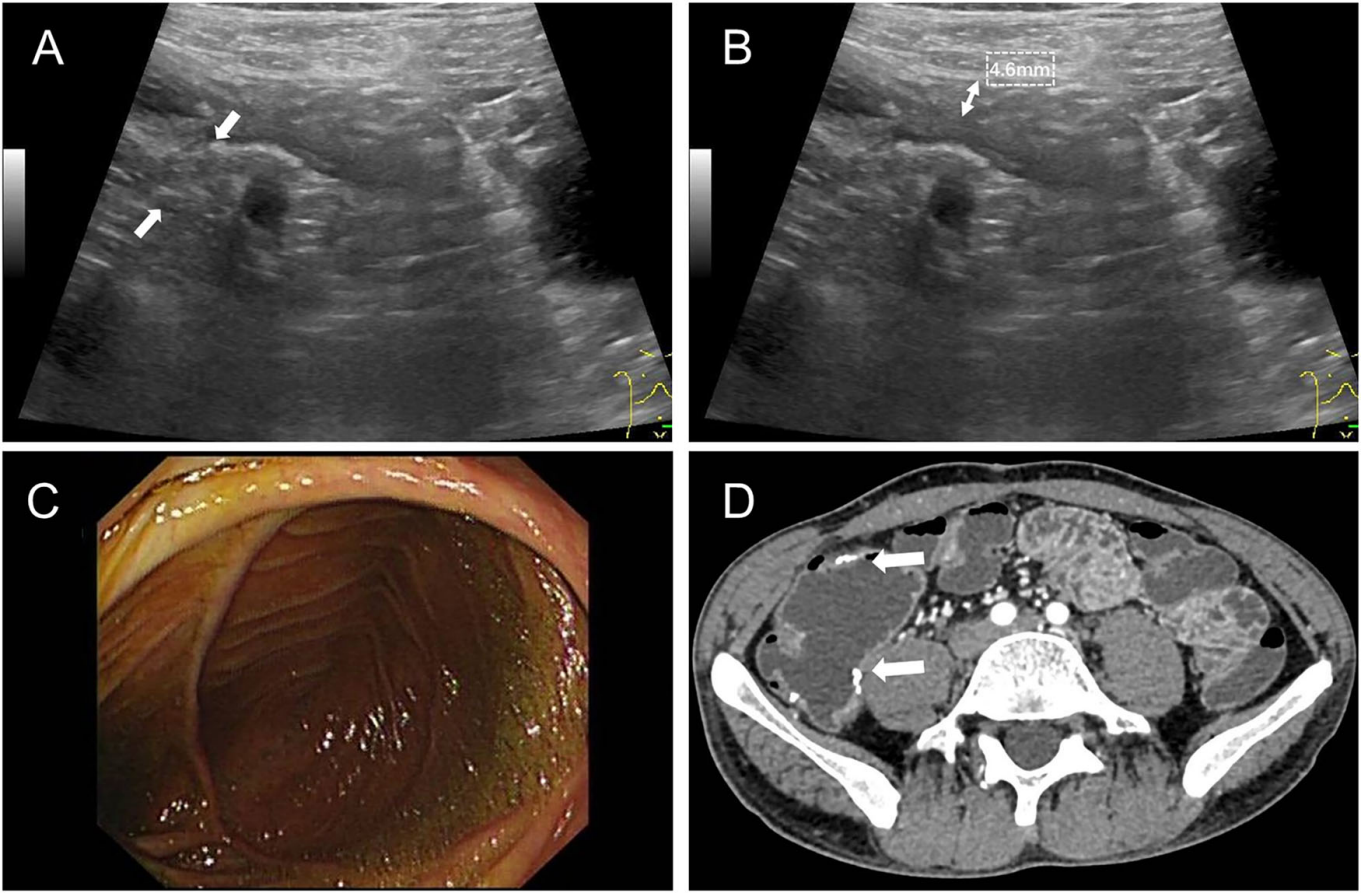
A 39-year-old male patient with CD presented with abnormal thickening of the intestinal wall without HMF. The mesenteric fat was normal, displaying striated, intermixed echoes adjacent to the bowel wall (**A**, solid arrow), and the thickness of the ileum measured 4.6 mm (**B**). One year after a partial enterectomy, no ulcers were found during endoscopy (**C**), and a CT scan showed that the bowel wall at the anastomosis (hollow arrow) was normal (**D**). CD, Crohn’s disease; HMF, hyperechoic mesenteric fat

The AUC for HMF in predicting EPR [0.808, 95% confidence interval (CI): 0.73–0.87] was significantly higher than that of BWT (0.618, *p* < 0.05) and abscesses/fistulas (0.599, *p* < 0.05; Table 3 and Fig. 2). Figures 3 and 4 show cases of early recurrence in patients with CD.

**Table 3:** Comparative predictive performance of IUS parameters (BWT, HMF, and abscess/fistula) for EPR

Variables	OR	95% CI	*p* value
BWT	1.241	1.046–1.474	0.014
HMF	21.250	8.280–54.539	< 0.001
Abscess/fistula	2.358	1.119–4.972	0.023

*BWT* bowel wall thickness, *HMF* hyperechoic mesenteric fat, *EPR* early postoperative recurrence

### Univariate and multivariate analyses of clinical characteristics and IUS parameters for predicting EPR

Univariate logistic regression analysis identified preoperative CRP, ESR, disease location (colonic), postoperative immunosuppressive therapy, and the IUS parameters HMF, abscess/fistula, and BWT as candidate predictors of EPR (all *p* < 0.05; Table 4).

**Table 4:** Univariate and multivariable analysis of parameters in predicting EPR

Variables	Univariate analysis			Multivariable analysis		
	OR	95% CI	*p* value	OR	95% CI	*p* value
**Clinical parameter**						
Gender	0.57	0.269–1.205	0.141			
Age	0.984	0.951–1.018	0.348			
Time from CD diagnosis to surgery	0.998	0.992–1.005	0.632			
BMI	0.942	0.815–1.089	0.420			
Type of surgical anastomosis						
Side-side	-	-	0.203			
End-side	2.292	0.629–8.346	0.209			
End-end	2.183	0.735–6.484	0.160			
Behavior of the disease						
Non-stricturing and non-penetrating	-	-	0.302			
Stricturing	2.000	0.711–5.626	0.189			
Penetrating	2.148	0.792–5.825	0.133			
Perianal disease	1.074	0.530–2.174	0.844			
Smoking history	0.000	0.000–∞	0.999			
Type of disease						
Ilea	-	-	-			-
Colonic	4.444	1.066–18.528	0.041	1.838	0.220–15.366	0.574
Ileocolonic	1.088	0.502–2.358	0.831	0.861	0.292–2.541	0.786
Previous intestinal resection	1.454	0.532–3.973	0.465			
Preoperative CRP	1.021	1.009–1.034	0.001	1.014	0.997–1.030	0.100
Preoperative ESR	1.033	1.009–1.058	0.007	1.018	0.986–1.051	0.267
Preoperative PLT	1.001	0.996–1.006	0.763			
Preoperative albumin	0.985	0.932–1.042	0.599			
Preoperative medications						
5-ASA	1.021	0.424–2.458	0.963			
Steroid	0.770	0.287–2.068	0.604			
Immunosuppressants	1.541	0.749–3.171	0.240			
Biologics	2.217	0.855–5.746	0.101			
Postoperative medications						
5-ASA	0.250	0.051–1.229	0.088			
Steroid	3.566	0.691–18.411	0.129			
Immunosuppressants	2.514	1.142–5.534	0.022	1.678	0.564–4.994	0.352
Biologics	0.620	0.303–1.266	0.189			
**Ultrasound parameter**						
BWT	1.241	1.046–1.474	0.014	1.026	0.820–1.285	0.822
BWS						
Preserved	-	-	0.134			
Focal disappeared	1.867	0.819–4.252	0.137			
Extensive disappeared	2.618	0.969–7.073	0.058			
Limberg classification						
Grade 0	-	-	0.895			
Grade I	1938569811.421	0.000–∞	1.000			
Grade II	1.200	0.121–11.865	0.876			
Grade III	0.857	0.229–3.203	0.819			
Grade IV	1.283	0.353–4.661	0.705			
HMF	21.250	8.280–54.539	< 0.001	18.810	6.459–54.775	< 0.001
Abscess/fistula	2.358	1.119–4.972	0.024	1.435	0.512–4.019	0.492
Mesenteric lymphadenopathy	2.098	0.914–4.817	0.081			
Abdominal effusion	2.291	0.404–12.993	0.349			

*Non-EPR* non-early postoperative recurrence, *EPR* early postoperative recurrence, *CRP* C-reactive protein, *ESR* erythrocyte sedimentation rate, *PLT* platelet, *5-ASA* 5-aminosalicylic acid, *BWT* bowel wall thickness, *BWS* bowel wall stratification, *HMF* hyperechoic mesenteric fat

These candidate variables were subsequently entered into a multivariate logistic regression model. HMF emerged as an independent predictor of EPR (odds ratio (OR) = 18.810, 95% CI: 6.459–54.775; *p* < 0.001) (Table 4).

## Discussion

IUS is a well-established, patient-friendly tool for managing CD [6]. However, its role in predicting EPR—a major clinical challenge typically assessed by invasive endoscopy—is not well defined. This study demonstrates that the imaging findings of IUS, including HMF, BWT, and abscess/fistula in CD patients, were associated with EPR. The multivariate logistic regression model showed that only HMF was an independent predictor of EPR; the AUC value was 0.808 (0.728–0.873).

Successful risk stratification and appropriate perioperative management were key to preventing post-operative recurrence in CD patients [12]. In the present study, 62 of 110 (56.4%) patients developed early recurrence within 1 year after surgery, which was similar to the rate of 30–90% reported in other studies [2, 3]. Previous studies have shown correlated factors that increased the risk of EPR, such as disease behavior, smoking, resection length, and previous resection history and serology [7, 11]. Our study showed that elevated preoperative CRP, ESR, disease location (colonic), and postoperative immunosuppressive therapy were clinical predictors of EPR, which was partially consistent with previous studies. Notably, while immunosuppressants and biologics (particularly the latter) have established efficacy in CD [13–15], this effect was not observed in our study. Two factors may explain this discrepancy: first, as a retrospective study, variability in clinicians’ postoperative biologic prescribing patterns may have introduced selection bias, as treatment was not standardized based on recurrence risk. Second, the study’s long timeframe includes periods before 2020, when biologic accessibility and affordability in China were limited, and some patients had previously failed biologic therapy (e.g., infliximab). As this study focused primarily on IUS, we emphasized the role of sonographic predictors—rather than pharmacological interventions—in assessing recurrence risk.

Accumulating evidence indicates that visceral adipose tissue, particularly IMF, significantly influences CD progression and long-term outcomes through immunomodulatory and endocrine functions [7, 9]. Mechanistically, IMF compartmentalizes inflammatory processes by regulating host responses to bacterial translocation [9, 16], while concurrently impairing mucosal drug delivery. This correlates with complex disease behavior, suboptimal treatment efficacy, and transmural inflammation [7]. Notably, HMF, detected by IUS, serves as a noninvasive imaging biomarker reflecting these underlying pathological changes of IMF.

Research on sonographic evaluation of mesenteric fat and its correlation with EPR in CD remains limited. According to the expert IUS consensus, HMF manifests as hypertrophic mesenteric encasement surrounding thickened bowel walls, characterized by hyperechogenicity, loss of fat lobulation, and mass-like wrapping—distinct from the striated, mixed-echogenicity pattern of normal mesentery [6]. Using this qualitative assessment method, we observed significantly higher EPR probability in HMF-positive patients. Specifically, HMF prevalence was markedly greater in the EPR group vs the non-EPR group (86% vs 23%; *p* < 0.001).

Conventional ileocolic resection involves bowel resection along the mesenteric border while preserving the mesentery. Residual inflamed fat and vessels within the preserved mesentery may perpetuate inflammation and drive disease recurrence [17]. Notably, HMF demonstrated superior predictive value for EPR (AUC = 0.808) compared to other IUS parameters such as BWT or abscesses/fistulas. Emerging evidence indicates that mesenteric excision in CD surgery is feasible and safe, potentially reducing recurrence rates [18, 19]—supporting HMF as a key modifiable risk factor. This underscores the clinical utility of HMF assessment for identifying high-risk patients requiring intensified surveillance and prophylactic therapy.

This study has several limitations. First, this single-center retrospective analysis with a limited sample size necessitates validation in prospective cohorts. Second, heterogeneity in surgical techniques, surgeon expertise, and evolving therapeutic paradigms over the extended study period may have introduced confounding biases, potentially affecting patient outcomes. Third, due to non-standardized surveillance intervals, the time to the first positive assessment may not accurately represent the true onset of recurrence, limiting conclusions on optimal monitoring timing.

In summary, the preoperative IUS parameter HMF serves as a significant predictor of EPR risk in CD patients. As a readily accessible ultrasonographic biomarker, HMF enables clinicians to identify high-recurrence-risk patients within the first year after intestinal resection, facilitating intensified surveillance and proactive management.

## Supplementary information

ELECTRONIC SUPPLEMENTARY MATERIAL

## Data Availability

The datasets used and/or analyzed during the current study are available from the corresponding author upon reasonable request.

## Abbreviations

IMF: Inflammatory mesenteric fat
BWS: Bowel wall stratification
BWT: Bowel wall thickness
CD: Crohn’s disease
CI: Confidence interval
CRP: C-reactive protein
ESR: Erythrocyte sedimentation rate
HMF: Hyperechoic mesenteric fat
IUS: Intestinal ultrasound
