# Supplementary material for: Hyperechoic mesenteric fat on preoperative intestinal ultrasound predicts early postoperative recurrence in Crohn’s disease
Source: Insights Imaging. 2026 Apr 16;17:100. doi: 10.1186/s13244-026-02251-2 (PMC13087000; doi:10.1186/s13244-026-02251-2)
Supplement: Supplementary file 1 — ELECTRONIC SUPPLEMENTARY MATERIAL [file 13244_2026_2251_MOESM1_ESM.pdf]

Hyperechoic mesenteric fat on preoperative intestinal ultrasound predicts early postoperative recurrence in Crohn's disease

ELECTRONIC SUPPLEMENTARY MATERIAL

Supplementary Table 1. Inter-observer agreement for the IUS parameters

Variables	Agreement level<br>Kappa value or ICC
HMF	0.886#
BWT	0.905*
BWS	0.779#
Limberg classification	0.788#
Abscess/fistula	0.804#
Mesenteric lymphadenopathy	0.721#
Abdominal effusion	0.468#

HMF, hyperechoic mesenteric fat; BWT, bowel wall thickness; BWS, bowel wall stratification; # Data are presented with kappa value for categorical variables; * Data are presented with intraclass correlation coefficient (ICC) for continuous variables.
